# Vacuole histomorphology characteristics and their same immunophenotype with the gut wall: A histological study using human fetuses

**DOI:** 10.3389/fped.2026.1915639

**Published:** 2026-10-02

**Authors:** Xuelai Liu, Zhe-Wu Jin, Yuqiang Chen, Mao Ye, Feng He, Xiaoyun Cui, Huan Wang, Chaosheng He

**Affiliations:** 1 Department of General Surgery, Capital Center for Children’s Health, Capital Medical University, Capital Institute of Pediatrics, Beijing, China; 2 Department of Anatomy, School of Basic Medical Sciences, Jilin Medical University, Jilin, China; 3 Department of Biochemistry and Immunology, Capital Center for Children's Health, Capital Medical University, Capital Institute of Pediatrics, Beijing, China; 4 Department of Science and Technology, Capital Center for Children's Health, Capital Medical University, Capital Institute of Pediatrics, Beijing, China; 5 Department of Pathology, Beijing Children’s Hospital Heilongjiang Hospital, Harbin, China; 6 Department of Pediatric Surgery, The First Affiliated Hospital of Xiamen University, Xiamen, China

**Keywords:** compartment, gut wall, immunophenotype, mucosal epithelium, vacuoles

## Abstract

**Background:**

Intestinal atresia (IA) has been associated with abnormal embryonic intestinal recanalization, a developmental process in which transient vacuoles form and merge with the developing mucosa. This study aimed to investigate the histomorphological characteristics of developing vacuoles and the immunophenotypic similarities between developing vacuoles and the gut wall.

**Methods:**

Serial sagittal sections from human fetuses of estimated gestational age (GA) of 8–12 weeks were examined by hematoxylin and eosin (H&E) staining and immunohistochemistry for CK19, CK5/6, and vimentin.

**Results:**

A total of eight human fetuses were included in this study. In fetuses at GA 8 weeks, HE staining showed that vacuoles were divided into two components: a peripheral basal compartment and a central luminal compartment, which were separated by a basal membrane. The basal compartment contained a single layer of columnar epithelial cells located on the basal membrane, whereas the luminal compartment contained scattered cells. Few histological changes were observed in fetuses at GA 10 weeks. Immunohistochemical staining showed cytokeratin 19- and cytokeratin 5/6-positive cells in both vacuolar epithelia and gut mucosal epithelium, and they were present continuously, whereas the vimentin-positive cells were present in the luminal compartment of vacuoles, as well as the lamina propria and submucosa of the gut wall.

**Conclusion:**

Vacuoles comprise both mesenchymal cells morphologically in the central luminal compartment and epithelial cells in the peripheral basal compartment. The basal compartment of vacuoles and gut mucosal epithelium have the same epithelial immunophenotype; the luminal compartment of vacuoles and the lamina propria and submucosa of the gut wall have the same mesenchymal immunophenotype.

## 1 Introduction

Intestinal atresia (IA) is a congenital intestinal malformation and a frequent cause of neonatal intestinal obstruction. Etiologically, IA is associated with defects in embryonic recanalization at gestational age (GA) 8–12 weeks (1–3). Our previous study has shown the process of intestinal lumen formation in human embryos and fetuses and the morphological mechanism through histological examination and 3D reconstruction, by which defective intestinal lumen formation led to IA (4). Moreover, through the evaluation of differential lumen development from the proximal to the distal intestine, we have revealed that, at GA 6–7 weeks, the embryonic gut was filled with mesenchymal cells and no lumen was noted, whereas, at GA 9–10 weeks, the gut lumen containing numerous vacuoles of different sizes had formed, with some vacuoles merging with the developing mucosal epithelium (4, 5). Our examination of intestinal septal specimens has shown that the residual vacuoles arising from human embryonic gut were surrounded by fibrous tissue in the submucosal layer in septa of IA patients (6). These findings indicate that vacuoles may play a significant role in both intestinal development and pathogenesis of IA. However, the morphological characteristics of typical vacuoles during gut development have not yet been described. Furthermore, it is still unclear whether the developing vacuoles in the mesenchyme express the same cell immunological markers as the developing mucosal epithelium, or, in other words, whether these vacuoles merging into the gut wall in the mesenchyme and the developing mucosal epithelium have the same immunophenotype.

To address this question, epithelial and mesenchymal markers can be used to compare developing vacuoles with adjacent gut-wall compartments. Cytokeratins (CK) are a family of at least 20 intermediate filament proteins found in epithelial cells. These proteins are located throughout cells, from the nucleus to cell junctions, and function as scaffolds to maintain cellular integrity and internal cellular transport (7). Different types of epithelia contain up to six specific CK subtypes (8), with CK19 mainly found in columnar epithelial cells in the intestinal mucosa. Although CK19 has also been reported to be expressed in gut mucosal epithelium of human fetuses (9), so far it is not clear whether CK19 or other CK family members are expressed in vacuoles in the mesenchyme of the developing gut and in those vacuoles merging into the gut wall.

The present study sought to investigate the morphological characteristics of vacuoles during their development. In addition, this study sought to determine whether epithelial (CK19 and CK5/6) and mesenchymal (Vimentin) biomarkers are expressed by vacuoles in the mesenchyme of the developing gut and in those merging into the gut wall, through which we assessed their immunophenotype in both the vacuoles in the mesenchyme (or merging into the gut wall) and developing mucosal epithelia.

## 2 Materials and methods

### 2.1 Chemicals and reagents

Double-distilled water was prepared in-house. Phosphate-buffered saline (PBS), paraformaldehyde, citrate solution, and ethanol solutions of different concentrations were purchased from Solarbio (China). The normal goat serum was purchase from Dako Bioresearch (USA). Primary antibodies are listed in Table 1. HRP-conjugated secondary antibody (Santa Cruz Biotechnology, CA, USA) and a DAB (3,3′-diaminobenzidine tetrahydrochloride) detection kit (Zhongshan Golden Bridge, China) were used for immunohistochemistry. H&E staining solutions (Sigma, USA) were used for histological morphology.

**Table 1:** Primary antibodies used for immunohistochemical characterization of epithelial and mesenchymal immunophenotypes in developing human fetal intestinal vacuoles and gut wall.

Antigen	Abbreviation	Dilution	Species	Manufacturer
Vimentin	Vimentin	1:200	Rabbit	Abcam
Cytokeratin5/6	CK5/6	1:150	Mouse	Abcam
Cytokeratin19	CK19	1:200	Rabbit	Abcam

### 2.2 Fetal specimens

The study was performed in accordance with the Declaration of Helsinki. Aborted human fetal specimens with estimated GA 8–12 weeks were examined. Gestational age was estimated from crown-rump length (CRL), a well-established correlate of embryonic and fetal age (4–6, 10–13). The use of human tissues complied with the principle of parental informed consent and this study was approved by the Medical Ethics Committee of the School of Basic Medical Sciences, Jilin Medical University (JNU20190318IRB62), and the Capital Institute of Pediatrics (SHERLLM2023054; SHERLL2022047).

### 2.3 Histologic analysis

Histological analysis was performed as described previously (4, 5). Briefly, fetal specimens were fixed in 10% formalin for 24 h at 4° C, dehydrated through a graded alcohol series, cleared in xylene, embedded in paraffin, and cut into 5 µm serial sagittal sections. Sections were mounted on TESPA-coated glass slides, deparaffinized, rehydrated, and stained with hematoxylin and eosin (H & E). After staining, sections were dehydrated in ethanol, cleared in xylene, mounted in neutral balsam, and examined by light microscopy (Olympus SZX7 and Nikon Eclipse E600, Japan). Slides were digitally scanned using a 3DHISTECH scanner (3DHISTECH, Hungary). Jejunal regions were identified on sagittal sections, and comparable anatomical regions from the central abdominal cavity were evaluated across fetuses.

### 2.4 Immunohistochemistry

The method used for immunohistochemical staining was the same as described previously (14–17). Briefly, the sections of 5 μm thickness were deparaffinized and rehydrated, and endogenous peroxidases were quenched by incubation in 3% hydrogen peroxide in methanol for 5–10 min. Following antigen retrieval, the sections were subsequently incubated with blocking solution containing 5% normal goat serum for 1 h at room temperature. The sections were incubated with primary antibodies (Table 1) overnight at 4° C in the dark, were washed in phosphate-buffered saline (PBS), and were incubated with HRP-conjugated secondary antibody for 2 h. The sections were subsequently incubated with DAB (3,3′-diaminobenzidine tetrahydrochloride) to visualize antibody binding and were counterstained with hematoxylin.

## 3 Results

### 3.1 Temporal morphological characteristics of vacuoles during GA 8–12 weeks

Eight human fetuses were included: two with CRL 18–20 mm (approximately GA 8 weeks), three with CRL 31–36 mm (approximately GA 10 weeks), and three with CRL 85–95 mm (approximately GA 12 weeks). In the present study, we investigated the morphological characteristics of vacuoles during GA 8–12 weeks. At GA 8 weeks, HE staining showed the presence of primitive gut epithelium and relatively low villi (Figures 1A,B). Vacuoles were typically densely packed and clustered within the primitive gut lumen, whereas developing villi appeared as protruding mucosal structures. At sites where vacuoles merged with the developing mucosal epithelium, well-formed villous protrusions were absent or poorly developed. Cells were distributed throughout the lamina propria of the gut mucosa, with narrow gaps among vacuoles (Figure 1B). Furthermore, vacuoles were noted to be divided into two components in HE staining: a peripheral basal compartment and a central luminal compartment, which were separated by a basal membrane. The basal compartment contained a single layer of columnar epithelial cells located on the basal membrane, whereas the luminal compartment contained scattered cells (Figure 1C). The nuclei in the epithelial cells were arranged in a disorderly fashion, with typical polarization not observed. In addition, goblet cells were not noted in the columnar epithelium during this developmental stage (Figures 1B,C).

**Figure 1:**
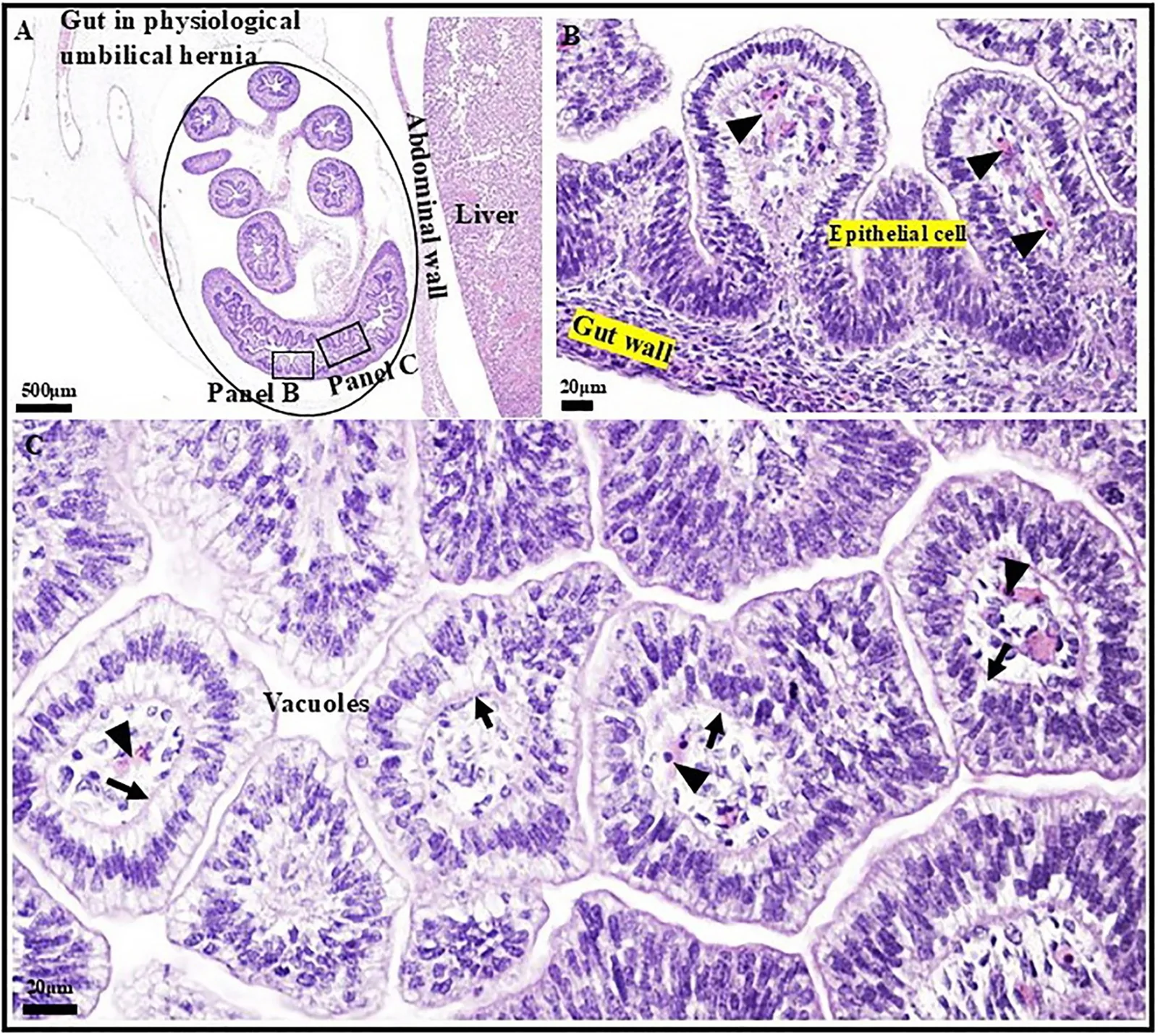
Histologic examination of human fetuses of gestational age 8 weeks. Sagittal sections of human fetuses of CRL 18–20 mm (approximate GA 8 weeks) were stained with H & E. **(A)** Developing gut located in the physiological umbilical hernia. **(B)** Gut mucosal epithelium. Low protruding villi and cells (black arrowhead) were distributed throughout the lamina propria of the gut mucosa, with the nuclei in the columnar epithelial cells arranged in a disorderly manner. **(C)** Developing vacuoles in the gut. Vacuoles consisted of basal and luminal compartments, separated by a basal membrane (black arrow). Scattered cells were present in the luminal compartment (black arrowhead).

By GA 10 weeks, the inner circular muscle had been found to develop in the gut wall (Figures 2A,B). The villi of the mucosa in the gut wall protruded, and canalization of the gut was further enhanced (Figure 2B). HE staining showed that, compared with the columnar epithelium of vacuoles at GA 8 weeks, the vacuolar epithelium located on the basal membrane was relatively flat in this developmental stage; moreover, the nuclei in epithelial cells were arranged in a disorderly fashion, and no polarization was noted (Figure 2C). Goblet cells were noted to differentiate among the columnar epithelial cells in both the epithelium of vacuoles and the gut mucosal epithelium. Scattered cells could still be seen in the luminal compartment of vacuoles at this developmental stage (Figures 2B,C).

**Figure 2:**
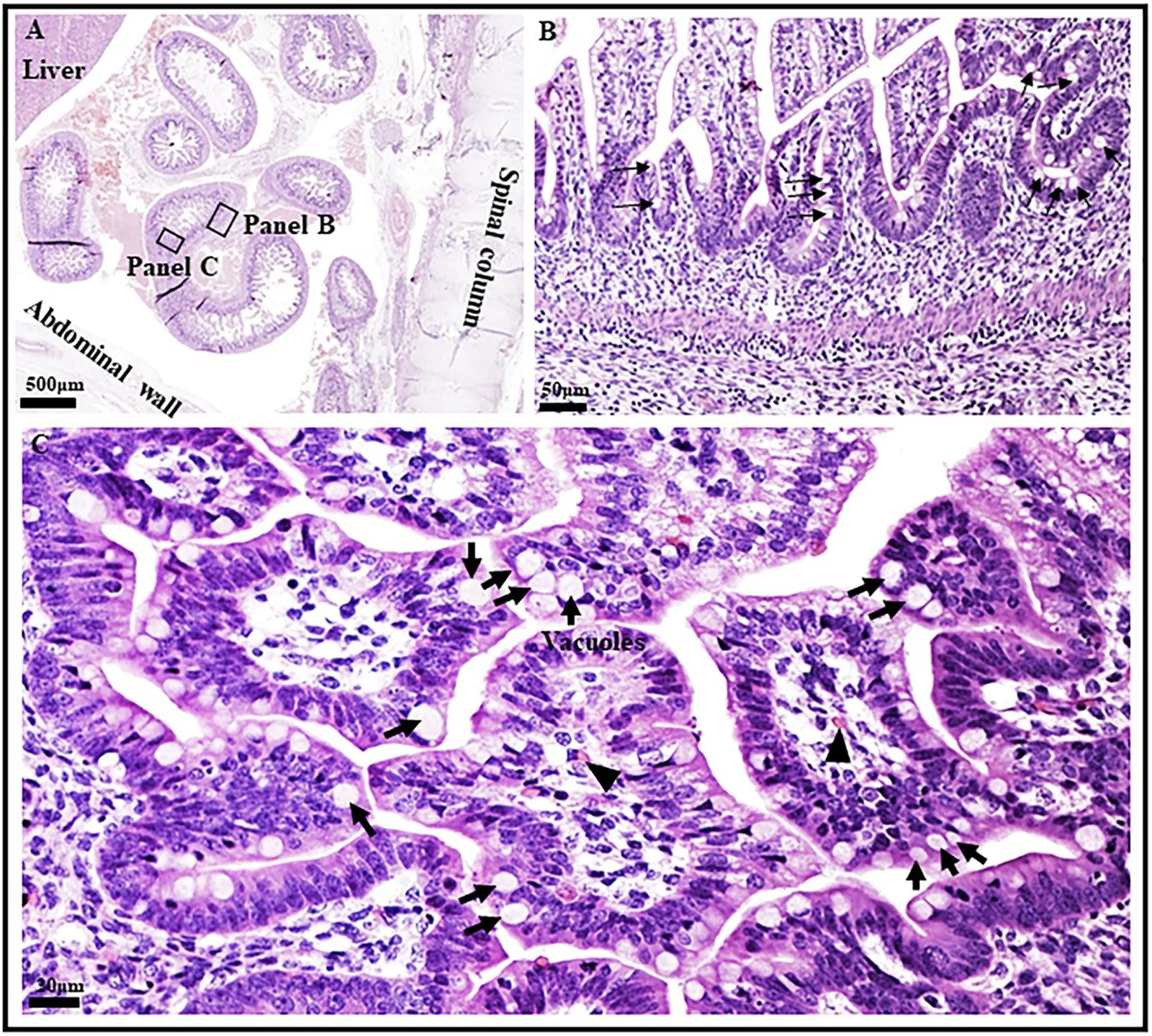
Histologic examination of human fetuses of gestational age 10 weeks. Sagittal sections of human fetuses of CRL 31–36 mm (approximate GA 10 weeks) were stained with H & E. **(A)** Developing gut in the abdominal cavity. **(B)** Gut mucosal epithelium. Obvious protrusion of low villi, with goblet cells (black arrow) distributed throughout the columnar epithelial cells in the gut mucosal epithelium. **(C)** Developing vacuoles in the gut. The vacuolar epithelium became relatively flat, with no polarization observed. Scattered cells (black arrowhead) and differentiated goblet cells were present in the luminal compartment (black arrow).

At GA 12 weeks, both the inner circular muscles and outer longitudinal muscles in the gut wall developed, and canalization of the gut was further enhanced (Figures 3A,B). The vacuoles had shriveled, with typical single-layer epithelial cells being present in the basal compartment of the vacuoles under HE staining (Figure 3C). The nuclei of epithelial cells were arranged in an orderly fashion and were close to the basal membrane; the vacuolar epithelium was polarized. Goblet cells were present among the single-layer epithelial cells in both the vacuoles and gut mucosa (Figures 3B,C).

**Figure 3:**
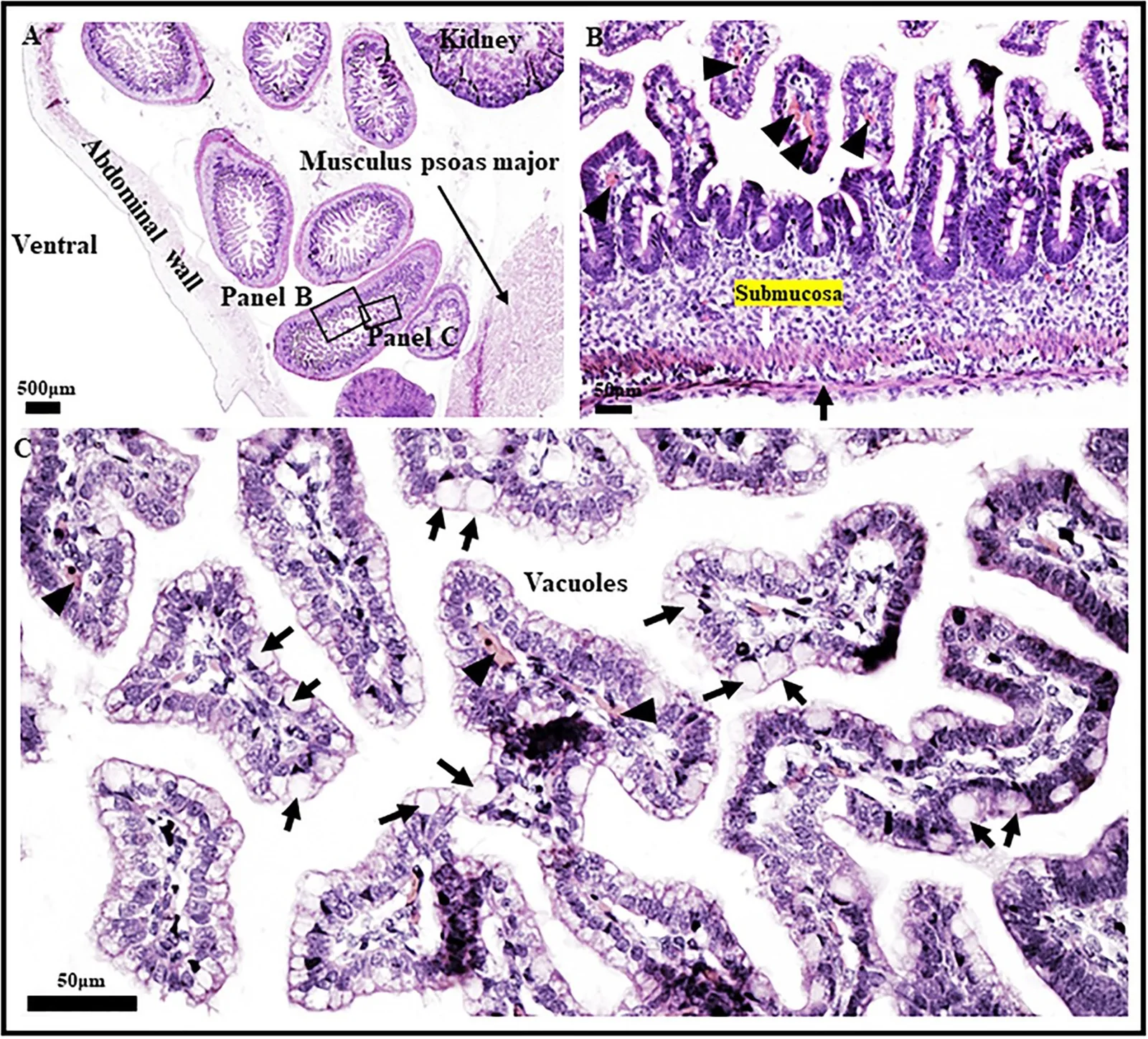
Histologic examination of human fetuses of gestational age 12 weeks. Sagittal sections of human fetuses of CRL 85–95 mm (approximate GA 12 weeks) were stained with H & E. **(A)** broad gut lumen was observed at this stage. A. Developing gut in the abdominal cavity. **(B)** Gut mucosal epithelium, showing an inner circular muscle (white arrow) and outer longitudinal muscles (black arrow). Goblet cells and scattered cells (black arrowhead) were observed in both the gut mucosal epithelium and developing vacuoles. **(C)** Mature vacuoles in the gut, showing single-layer squamous epithelium in vacuoles and nuclear polarization. Scattered cells (black arrowhead) and differentiated goblet cells (black arrow) were present in the luminal compartment.

### 3.2 Mesenchymal immunophenotype was found in the luminal compartment of vacuoles

We next detected whether the scattered cells in the luminal compartment of vacuoles were mesenchymal immunophenotype cells. In order to determine this, sections from fetuses at GA 10 weeks were incubated with an antibody to vimentin, a marker of the mesenchymal phenotype. Immunohistochemical staining showed that cells in the luminal compartment were positive for vimentin expression, indicating that those scattered cells in the luminal compartment of vacuoles were mesenchymal immunophenotype cells (Figures 4A–C). In addition, the lamina propria and submucosa of the gut wall were positive for vimentin at this developmental stage (Figure 4D).

**Figure 4:**
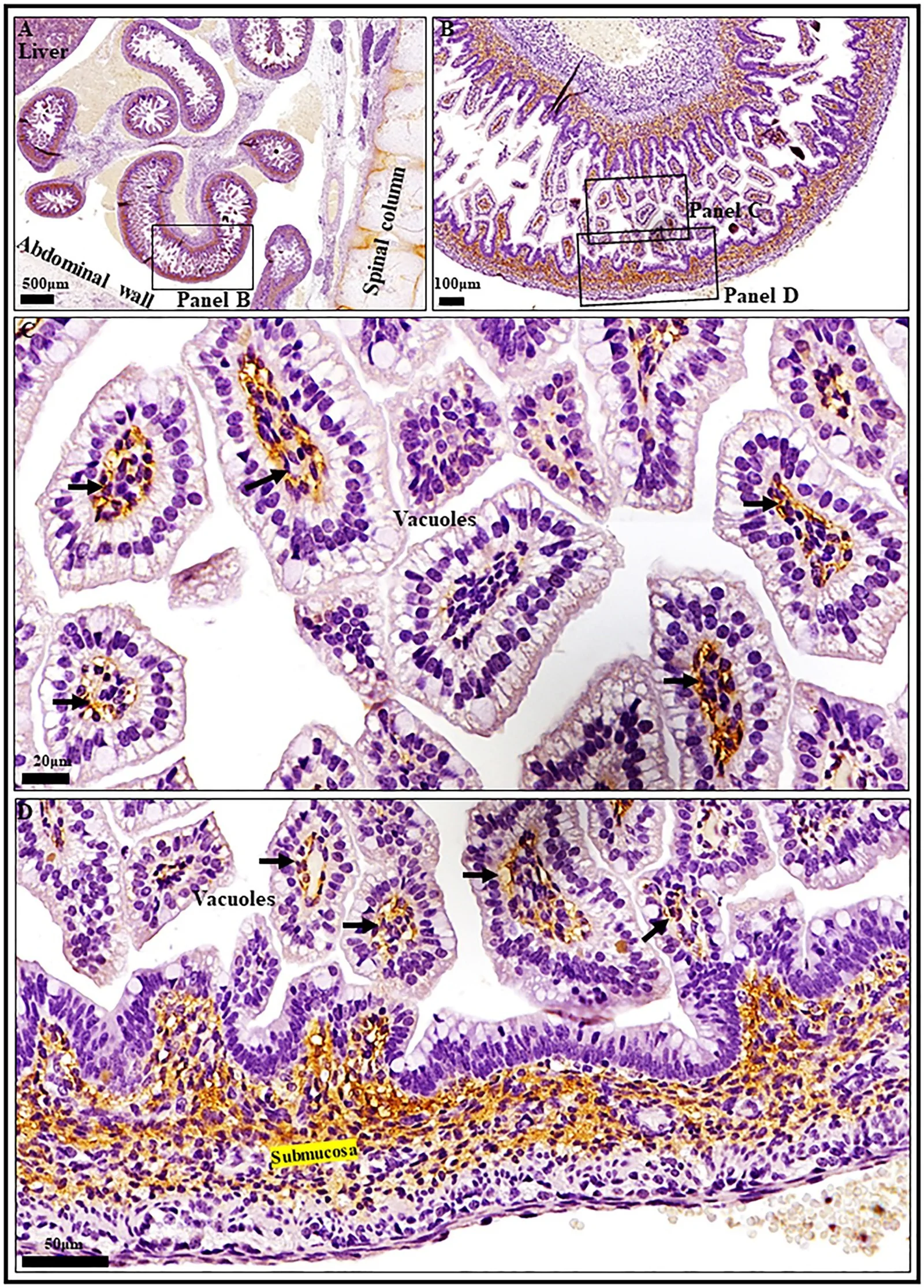
Vimentin expression in human fetuses of gestational age 10 weeks. Sagittal sections of human fetuses of CRL 31–36 mm (approximate GA 10 weeks) were stained with an antibody to vimentin. **(A,B)** Developing gut in the abdominal cavity. **(C)** Vimentin-positive cells (black arrow) in the luminal compartment of vacuoles. **(D)** Vimentin-positive cells in the lamina propria and submucosa of the gut wall.

### 3.3 Epithelial immunophenotype was noted in the vacuolar epithelia

We adopted CK19 as the biomarker to investigate the immunophenotype in the vacuolar epithelia. Immunohistochemical staining showed that vacuoles in the gut mesenchyme and epithelium in the basal compartment of vacuoles in the gut mesenchyme were positive for CK19 (Figures 5A–D). Further evaluation of CK19 expression in gut mucosal epithelium showed that CK19 expression was lower in mucosal crypts (Figure 5E). CK19 expression was continuous in both merging vacuoles of the gut wall and gut mucosal epithelium, suggesting the same epithelial immunophenotype between the vacuolar epithelium and gut mucosal epithelium during gut development. Furthermore, CK19 expression was observed in the mesentery and serosa at this developmental stage (Figures 5F–H).

**Figure 5:**
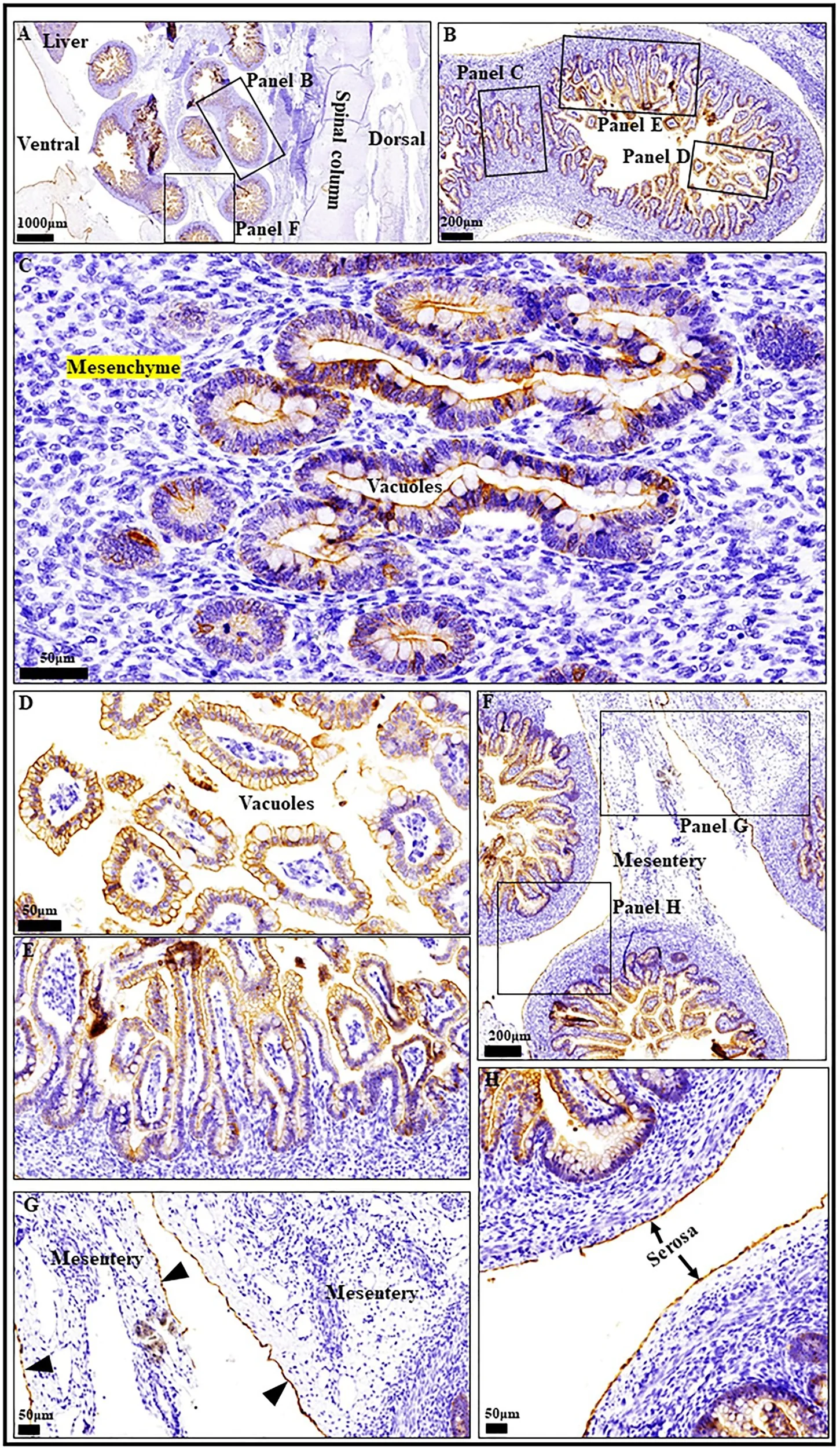
CK19 expression in human fetuses of gestational age 10 weeks. Sagittal sections of human fetuses of CRL 31–36 mm (approximate GA 10 weeks) were stained with an antibody to CK19. **(A,B)**. Developing gut in the abdominal cavity. **(C)** CK19-positive cells in vacuolar epithelium located in gut mesenchyme. **(D)** CK19-positive cells in epithelium in the basal compartment of vacuoles. **(E)** CK19-positive cells in merging vacuoles and gut mucosal epithelium, with CK19 expression being lower in mucosal crypts. **(F)** Developing mesentery around the gut. **(G)** CK19-positive expression in mesentery. **(H)** CK19-positive expression in the serosa of the gut.

As CK5/6 is specifically expressed in the epithelium of basal compartments, we further evaluated CK5/6 expression in the vacuolar and gut mucosal epithelium. Immunohistochemical staining showed that vacuoles in the gut mesenchyme and epithelium in the basal compartment of vacuoles were positive for CK5/6 expression (Figures 6A–D). The relatively lower CK5/6 expression in mucosal crypts was noted in gut mucosal epithelium (Figure 6E), and again, CK5/6 expression in both the merging vacuoles of the gut wall and gut mucosal epithelium presented continuous characteristics, confirming the same epithelial immunophenotype between vacuolar epithelium and gut mucosal epithelium (Figure 6).

**Figure 6:**
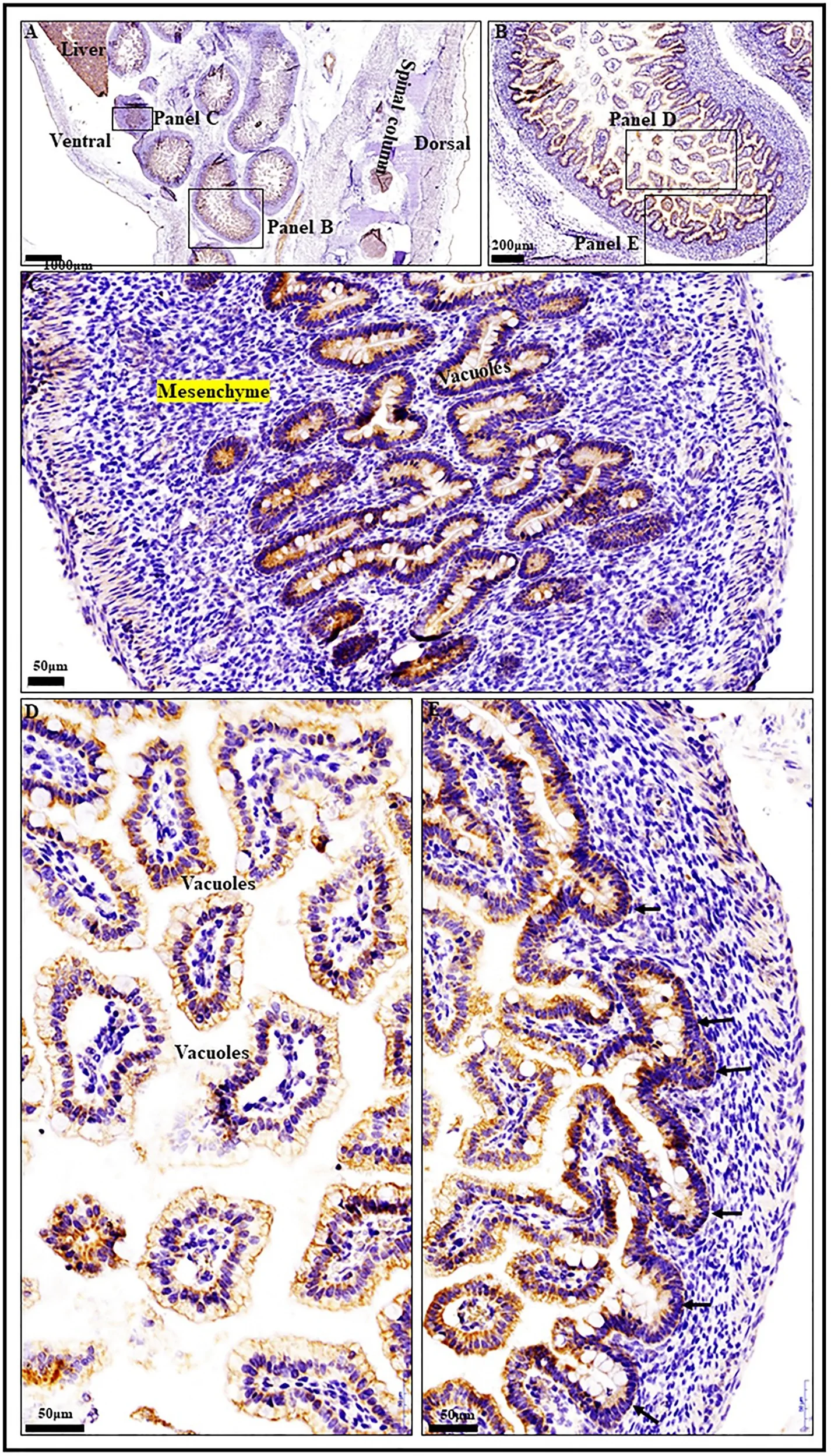
CK5/6 expression in human fetuses of gestational age 10 weeks. Sagittal sections of human fetuses of CRL 31–36 mm (approximate GA 10 weeks) were stained with an antibody to CK5/6. **(A,B)**. Developing gut in the abdominal cavity. **(C)** The CK5/6-positive cells in the vacuolar epithelium located in the gut mesenchyme. **(D)** CK5/6-positive cells in epithelium in the basal compartment of vacuoles. **(E)** CK5/6-positive cells in merging vacuoles and gut mucosal epithelium, with CK5/6 expression being lower in mucosal crypts.

## 4 Discussion

Although the exact etiology of IA is not completely understood, it has been linked to failure of recanalization of the intestinal duct and vascular dysplasia during the embryonic period (18, 19). Currently, failure of recanalization of the intestinal duct is widely regarded as the cause of IA (20–23). Under normal development, the embryonic intestine is obstructed by an epithelial plug at GA 5 weeks, followed by the appearance, expansion, and fusion of many vacuoles in the occluded lumen at GA 6–7 weeks and subsequent recanalization to form the intestinal lumen at GA 8–12 weeks (1–3). The primitive gut further undergoes dramatic elongation and forms a lumen lined by a single layer of epithelium during digestive organogenesis (24–26). Our previous study has shown the presence of residual vacuoles in the septum of fetuses with IA (4). Less is known, however, about the exact temporal morphological characteristics of vacuoles in the gut at GA 8–12 weeks. Moreover, although vacuoles have been found to merge with the developing mucosal epithelium, suggesting that these vacuoles were potentially cellular resources in the derivation and formation of mucosal epithelium during embryonic intestinal duct recanalization, there was insufficient histological evidence for the same immunophenotype between the developing vacuoles and the developing mucosal epithelium of the gut wall.

In the present study, we describe the temporal morphological characteristics of vacuoles in fetuses of GA 8–12 weeks. HE staining has shown that vacuoles are comprised of a basal compartment and a luminal compartment. During this developmental period, single-layer columnar epithelium changes toward squamous epithelium in the basal compartment, and the vacuoles become shriveled, which provides further evidence that vacuoles are only transient histological structures during gut development. Moreover, the nuclei of epithelial cells in the basal compartment, which are disordered at GA 8 weeks, become more orderly and polarized by GA 12 weeks. Beginning at GA 10 weeks, goblet cells start to differentiate in the epithelium of vacuoles, and scattered cells are observed in the luminal compartment of vacuoles during this developmental period. Furthermore, the epithelial villi in the lumen gradually become more protrusive, and the recanalization process becomes more complete, with more vacuoles merging into the gut wall.

The endoderm and mesoderm are involved in the development and formation of the primitive gut (27–29). Mucosal epithelium arises from endoderm, whereas the submucosal and muscular layers arise from mesoderm (30, 31). Interestingly, the present study shows that, at GA 8–12 weeks, some cells in the luminal compartment of vacuoles are surrounded by the basal compartment of vacuoles in the gut. We therefore assessed whether some cells in the luminal compartment of vacuoles were mesenchymal immunophenotype cells. Immunohistochemical staining showed that cells in the luminal compartment were positive for vimentin expression, indicating that they were mesenchymal immunophenotype cells. Although the lamina propria and submucosa of the gut wall were also positive for vimentin, we can only conclude that cells in the luminal compartment of vacuoles have the same immunophenotype as these gut-wall compartments. The above immunohistochemical staining could not confirm that during gut development, the vacuoles are composed of mesodermal cells, specifically mesenchymal cells in the luminal compartment of vacuoles.

The observation that CK19 is expressed in the mesentery and serosa further corroborates that CK19 is expressible on mesothelium-lined histological structures, and accordingly provides the basis for a clear interpretation of CK19 expression in the present study. The present study also revealed that vacuolar epithelium in gut mesenchyme and epithelium in the basal compartment of vacuoles, as well as gut mucosal epithelium, were positive for CK19 and CK5/6, biomarkers of intestinal epithelium, further indicating that they were epithelial immunophenotype cells. Accordingly, it is not confirmed that during gut development, the vacuoles are composed of endodermal cells, specifically epithelial cells in the basal compartment of vacuoles. Additional cell-lineage studies are needed if we aim to illustrate that both endoderm and mesoderm were constituents of gut vacuoles during gut development. Taken together with the fusion of vacuolar epithelium and gut mucosal epithelium during gut development that we found in a previous study (4), the same immunophenotype between the epithelium of the basal compartment of vacuoles and gut mucosal epithelium shown in this study, as well as the same immunophenotype between the luminal compartment of vacuoles and the lamina propria and submucosa of the gut wall, are potential indications that the vacuoles are cellular resources in the derivation and formation of mucosal epithelium during embryonic intestinal duct recanalization.

In summary, the present study describes the morphological characteristics of vacuoles during gut development and supplements current understanding of embryonic vacuoles. Furthermore, we revealed that developing vacuoles in the mesenchyme express the same immunophenotype as the gut wall, including mesenchymal and epithelial immunophenotypes, which suggests that the vacuoles merging into the gut wall in the mesenchyme and the developing mucosal epithelium may be homogeneous during gut development. This will be an important focus of our future studies. Owing to the scarcity and difficulty in obtaining human embryonic and fetal tissues, the present study could not include more specimens with multiple developmental stages. With the ongoing accumulation of embryos and fetuses in our team, we will further investigate the immunophenotypic features of vacuoles and gut wall across the different developmental stages, with the aim of advancing our understanding of the mechanisms underlying IA. This study is limited to descriptive analysis—a limitation we will address in future work by concentrating on fresh embryonic specimens (GA 8–12 weeks) for spatial transcriptomics, to investigate alterations in cellular spatial arrangement, as well as the regulatory molecules and signaling pathways that govern vacuole development during embryogenesis.

## Data Availability

The raw data supporting the conclusions of this article will be made available by the authors, without undue reservation.
